# Social and Economic Support for Mexican Older Adults Living With Dementia in Mexico and the United States

**DOI:** 10.1093/geroni/igaf122.600

**Published:** 2025-12-31

**Authors:** Emma Aguila, Mariana Lopez-Ortega, Rosa Garcia-Chanes, Jacqueline Angel

**Affiliations:** University of Southern California, Los Angeles, California, United States; National Institute of Geriatrics, Ciudad de Mexico, Estado de México, Mexico; National Institute of Geriatrics, Ciudad de Mexico, Estado de México, Mexico; The University of Texas at Austin, Austin, Texas, United States

## Abstract

Dementia is one of the most common causes of disability and dependence and is a frequent reason that older people require supportive living. In Mexico and the United States, Alzheimer’s and other dementias represent one of main cause of DALYS (disability-adjusted life-years) and it is estimated that there are approximately 1.6 million in Mexico and at least 6 million persons living with dementia in the U.S. However, the health systems are not prepared to adequately provide diagnosis, post-diagnosis care as well as care support at home for individuals living with dementia and their family caregivers. In addition, economic conditions of a large number of Mexican-origin older population are precarious given their working life in the informal sector or “gig economy” with limited access to employer provided benefits and social safety net policies in old age, relying primarily on family financial and in-kind support. The aim of this study is to investigate the burden of informal support in Mexico and the United States. We use the Mexican Health and Aging Study (MHAS) and the Health and Retirement Study (HRS) data sets linked to their Cognitive Aging Ancillary Study (Mex-Cog and HCAP) 2015-16. This allows for a detailed characterization of support received by individuals living with dementia and compares it with the support available and received by older adults with no impairment. The results allow for specific recommendations for further support programs and activities that focus on areas of need and are currently not covered either by public or family support.

